# Evaluation of Ebola virus disease surveillance system in Tonkolili District, Sierra Leone

**DOI:** 10.11604/pamj.supp.2019.32.1.14434

**Published:** 2019-01-21

**Authors:** Olayinka Stephen Ilesanmi, Olufunmilayo Fawole, Patrick Nguku, Abisola Oladimeji, Okoro Nwenyi

**Affiliations:** 1 Liberia Field Epidemiology and Laboratory Training Programme, Monrovia, Liberia; 2 Department of Epidemiology and Medical Statistics, Faculty of Public Health, University of Ibadan; 3 Nigeria Field Epidemiology and Laboratory Training Programme, Abuja, Nigeria

**Keywords:** Surveillance, Ebola Virus Disease, outbreak, Sierra Leone

## Abstract

**Introduction:**

The Ebola Virus Disease (EVD) epidemic devastated West Africa, with Sierra Leone recording over 50% of the 28,610 cases across the three most affected countries. Enhanced surveillance system was developed for improved identification of cases and response in Sierra Leone. Here, we evaluated the surveillance system to determine its strengths and challenges in meeting the set objectives.

**Methods:**

The EVD surveillance system in Tonkolili District, Sierra Leone, was assessed using the CDC updated guidelines for evaluating public health surveillance. In particular, the simplicity, stability, acceptability, flexibility, representativeness, sensitivity, positive predictive value and data quality of the system were assessed using EVD surveillance data and information from key informant interviews with program stakeholders.

**Results:**

The EVD surveillance system in Tonkolili District provided information and data on disease trends and outbreak report through official and rumours sources. Case definitions were well understood by participants, with willingness to continue surveillance activities after the EVD outbreak. Standardized data collection tools were in place and data communication was clear with feedback to surveillance units at all levels. The EVD surveillance was not operated within the Integrated Disease Surveillance and Response framework (IDSR). Data completeness was about 91%, consistency existed but data quality was poor (incompletely filled data and missing data existed). Regarding timeliness, samples arrived designated laboratory within 24 - 48 hours in 174 (84.9%). Sensitivity of the surveillance system was 88.5%. Predictive value positive was 25.8%. The stability was questionable since the government of Sierra Leone were not fully in charge of the system.

**Conclusion:**

While the simplicity of the EVD surveillance system in Tonkolili District facilitated its implementation, users suggested that the system did not meet expectations in terms of timeliness, flexibility and acceptability. There was a need to channel efforts towards integrating EVD surveillance into the IDSR. Data completeness and timeliness needed more attention. The District Health Management Team need to take ownership of the surveillance system for sustainability.

## Introduction

In 2013-2015, the West African region experienced its first major outbreak of Ebola Virus Disease (EVD) from December 2013, with intense transmission occurring in Guinea, Liberia and Sierra Leone. Sierra Leone had about 50% of the 28,610 cases across the three most affected countries [1]. In Sierra Leone, suspected, probable and confirmed EVD cases were 14,124 while 3,956 (28%) deaths were recorded. The EVD outbreak in West Africa was difficult to contain due to poor response capacity [2]. Once in the human population, the disease is easily transmitted through direct contact with infectious body fluids [3]. The average incubation period for most people who develop symptoms is about 10 days, with a range of 2-21 days. The case fatality rate ranges between 25-90% [1, 4]. Reduction in economic activities and further spread of other preventable diseases were reported during the EVD outbreak. This was due to the number of countries and people affected. The strategies identified in controlling the EVD outbreak included patient identification and isolation, contact tracing, safe patient and body transport systems, safe burial and environmental decontamination and community engagement activities [5]. Surveillance activities are important to bringing an end to infectious disease outbreak. Public health surveillance is the ongoing, systematic collection, analysis, interpretation and dissemination of data regarding a health-related event for use in public health action to reduce morbidity and mortality and to improve health [6]. Considering the importance of public health surveillance systems, evaluation should be conducted periodically. The outcome of the evaluation should include recommendations for improving quality and usefulness. The attributes evaluated in a surveillance system are simplicity, flexibility, data quality, acceptability, sensitivity, predictive value positive, representativeness, timeliness and stability [7]. The purpose of evaluating public health surveillance systems is thus to ensure that problems of public health importance are being monitored efficiently and effectively [8, 9]. During the post-outbreak period there is a need to put an ongoing system in place for the collection, analysis and interpretation of data which will be utilized in taking prompt decision. Therefore a surveillance system aims at ensuring early detection of a potential outbreak in order to institute prompt and effective control measures is essential. EVD was not part of the routine Integrated Disease Surveillance and Response (IDSR) in Sierra Leone prior to the 2013 outbreak. Enhanced surveillance was developed with increase human, material and financial resources for adequate response to detect response and protect against EVD without neglecting other diseases and conditions on the IDSR list. The enhanced surveillance was commenced in Tonkolili District, December, 2014. The enhanced surveillance system collects and analyses EVD data generated from the communities and health facilities in order to evaluate both the process and the outputs of the system. Here we conducted a systematic evaluation of the EVD surveillance system to recognize its successes as well as identify opportunities for enhancement, particularly in terms of system effectiveness for eliciting important alerts and identifying Ebola cases in the community.

## Methods

### Study area

Tonkolili District is in the Northern Province of Sierra Leone. Other districts in the Northern Province are Bombali, Port Loko, Kambia and Koinadugu. Tonkolili`s capital and largest city is Magburaka. The other major towns in Tonkolili include Mabonto, Bumbuna, Makali, Masingbi, Yele, Bendugu, Mile 91, Bumbuna, Yonibana and Matotoka. As of 2015, the district had a population of 531,435 [10]. The district occupies a total area of 7,003 km^2^ (2,704 sq mi) and comprises eleven Chiefdoms. Tonkolili District borders Bombali District to the northwest, Kono District to the east, Kenema District and Bo District to the southeast, Port Loko and Koinadugu Districts. Agriculture also plays a significant role in the economy. Tonkolili is strategically located in the centre of Sierra Leone. The district is crossed by many rivers including the Pampana River and Sierra Leone´s longest river, the Rokel. Tonkolili had 458 cases out of the 3,956 cases of EVD during the 2014-2016 outbreak in Sierra Leone [11]. All medical care is generally provided by a mixture of government, private and non-governmental organizations (NGOs). The Ministry of Health and Sanitation (MOHS) is responsible for health care. In Tonkolili, the medical facilities are 8 Community Health Centers (CHC), 9 Community Health Posts (CHP), 52 Maternal Child Health Posts (MCHP) and 1 government hospital, 2 mission clinics, 1 mission hospital, 1 NGO clinic and 1 private clinic. Traditional medicine forms part of the primary health care system in Sierra Leone.

### Surveillance stakeholders and attributes evaluated

The surveillance stakeholders at the district, community and health facility levels were interviewed. The stakeholders included the District Medical Officer of Health (DMO), an epidemiologist, a case investigator, contact tracers, data entry personnel, community leaders and an officer in charge of the health facility. In all, six health workers and 10 key informants were interviewed. We evaluated the operation of the system with respect to case definitions of EVD, resources for the surveillance system and reporting from both the community and health facility to the District Health Management Team (DHMT). The DHMT coordinates the District Ebola Response Centre (DERC). Attributes assessed included sensitivity, usefulness, stability, acceptability, representativeness, completeness, timeliness and data quality, in accord with the CDC updated guidelines for evaluating public health surveillance systems [7].

### Information flow in the EVD surveillance system

**Figure 1:**
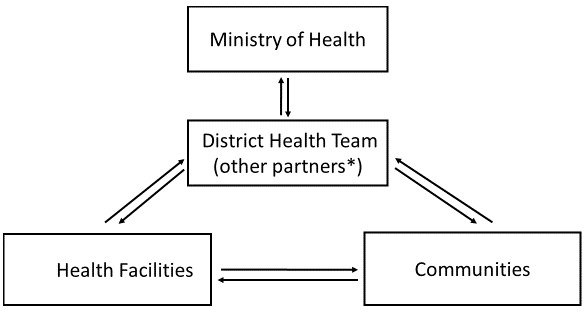
Information Flow in the EVD Surveillance System, Tonkolili District, Sierra Leone, December, 2014

**Figure 2:**
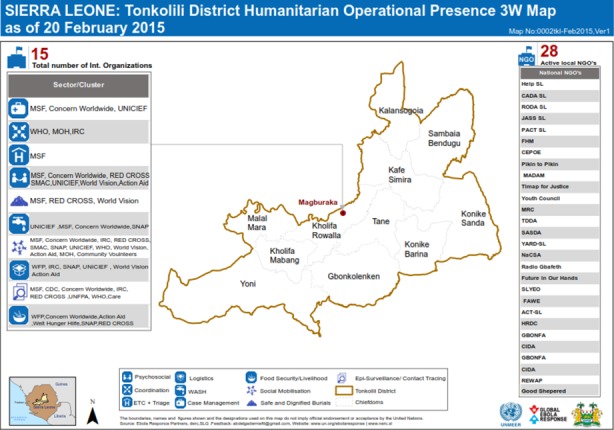
Partners involved in humanitarian services in Tonkolili District, Sierra Leone, December, 2014

Dissemination of information commences at the community level through any of contact tracers, burial team members, community heads, community representatives or any member of the community to the DHMT or health facility. The head of the health facility also sent data collected from the facility or the Ebola Treatment Units or the community to the District Health Team. The activities of other partners are coordinated form the district. The flow of data and information is shown in Figure 1, Figure 2 show the partners involved in the humanitarian mission [12].

### Assessment of qualitative attributes

Qualitative attributes assessed were simplicity, stability, acceptability, flexibility and representativeness. In the assessment of qualitative attributes, four questions (statements) were used for each attribute (Table 1). Three positive responses to the questions were categorised as excellent. Two positive responses to the questions were categorised as good, while below two positive responses were categorised as poor.

**Table 1:** Qualitative Attributes used in EVD Surveillance System Tonkolili District, Sierra Leone, December, 2014

Sn	Attributes	Questions
1	Simplicity	How easy is the case definition to understand?
		Is the reporting process clear and easy?
		Are cases immediately reported?
		How long does it take to fill the reporting forms (<5 minutes)?
2	Stability	Are notification forms available at the health facilities?
		Who manages the surveillance system?
		Are staff available to work on the surveillance system?
		Are computers available to process data generated
3	Acceptability	Is EVD part of the IDSR reporting system?
		Are health workers knowledgeable on EVD case definition?
		Are health workers knowledgeable on specimen collection?
		Are the community willing to report cases to the health facility?
4	Flexibility	Is change easy to introduce to the EVD Surveillance system?
		Is it easy to adapt changes in case based form?
		Is it easy adapt to changing information needs?
		How easy it is to adapt technological operating conditions with little additional time, personnel e g changing from paper based to electronic?
5	Representativeness	Are the reports generated by all the health facilities?
		Are the reports generated by all the communities?
		Does the data collection represent the state of EVD in the district?
		Is there a community feedback mechanism available?

### Assessment of quantitative attributes

Quantitative attributes assessed were sensitivity, positive predictive value and data quality (completeness and timeliness). To assess the quantitative attributes, retrospective review of records were conducted using the surveillance data collected from December 2014 to January 2015. The surveillance data used captured 247 people who had laboratory test done for EVD in Tonkolili District in December, 2015. The population under surveillance were the entire people leaving in Tonkolili District. Data was initially paper based and subsequently entered into excel spread sheet. Brief analysis of the data was done daily and the findings were shared during the debriefing meetings held daily with the DHMT. Data analysed was that of 247 people who were suspected of having EVD in the community using the community case definition. Their blood samples were collected and tested for EVD. Descriptive statistics was done. Age was described using median and range. A 2 by 2 table was constructed to determine sensitivity and positive predictive value. Data were analysed using SPSS for windows software version 21.

## Results

### List of all stakeholders and their roles in the surveillance system

The DMO was the head of the DHMT. He coordinated all district health activities. Case investigators were the first responders to all alerts. Contact tracers promptly identified any ill person in quarantine homes. Contacts were traced for a period of 21 days after last known interaction with any infected person. The data entry personnel collected data entry forms from the contact tracers’ supervisors on a daily basis. The community leaders complemented the efforts of the contact tracers in their respective community.

### EVD surveillance case definition for health facility

At health facility levels, three case definitions were used: 1) A **suspected** EVD case under investigation was defined as any person with or without known history of travel or stay in a country that had reported at least one confirmed case of EVD, within a period of 21 days before the onset of symptoms and who presented with sudden onset of high fever (> 38.0°C) and at least three of the following symptoms: headache, vomiting, diarrhoea, anorexia/loss of appetite, lethargy, stomach pain, aching muscles or joints, sore throat and difficult breathing. Inexplicable bleeding/haemorrhaging or inexplicable sudden death; 2) A probable case was a suspected case evaluated by a clinician or any deceased suspected case (where it had not been possible to collect specimens for laboratory confirmation) having an epidemiological link with a confirmed case; 3) A confirmed case was a suspected case with laboratory confirmation (positive IgM antibody, positive Polymerase Chain Reaction or viral isolation).

### Community case definition for Ebola Virus Disease

This simplified case definition was used during the outbreak response as part of the community alerting system. The community case definition was “any person who had unexplained illness with fever, diarrhoea, vomiting with or without bleeding which did not respond to antimalarial or who died after an unexplained severe illness with fever and bleeding”.

### Channels of communication

The level of integration with other systems was poor, due to the breakdown of the health system at the time of the surveillance evaluation, only few health facilities were functioning. Alert from the population (community) under surveillance was received through the alert desk at the District Ebola Response Centre (DERC). The DERC notified the case investigation teams. The teams contacted the contact tracing team and the ambulance team for follow up and movement of the patients respectively. The case investigation teams collected data on sick individuals while the contact tracers collected data on contacts. Laboratory data and case management data were collected at the Ebola Treatment Unit. Organisations that participated in data management were WHO, CDC, DERC and UNFPA teams. Dissemination of findings from each team members was done at the daily case investigators meeting, epidemiology and surveillance meeting, case management meeting and daily debriefing by the DHMT. At the DHMT all the representatives of the partners involved in the response met daily. Data confidentiality was maintained by ensuring that data was shared only with partners in the response.

### The resources used to operate the surveillance system

Several organizations were involved in the surveillance system. Each of the organisation was responsible for the provision of fund they require to function. It was difficult to ascertain all the resources that were utilized in the surveillance response. The list of the organisation included MSF, CDC, Africa Union, Concern Worldwide, IRC, RED CROSS, UNFPA, WHO, the British government and UNMEER. The manpower available for the activity included data managers, epidemiologist, contact tracers mentor and monitor. At least 3- 4 cars were available for use daily, training was conducted any time a new staff joined the Epidemiology and Surveillance pillar. Laptop computers were made available for daily use by all the partners. Internet facility was also provided by the US CDC. Provision of laboratory logistics was by the MSF. The African Union team provided decontamination services of EVD confirmed homes.

### Level of usefulness of the system

The surveillance system is useful in documentation of the distribution and spread of EVD. The system identified the chains of EVD transmission in the community. The system also empowered the communities to take action to stop chains of transmission. Other usefulness included improvement of health outcomes by increasing the timeliness in which EVD suspected cases were identified and received care and understanding of the structures in place to arrest the EVD outbreak. Description of the process of operation of the surveillance system and assessment of its key attributes and provision of appropriate recommendation to stakeholders for its improvement were part of its usefulness.

### Description of the EVD surveillance system attributes

Simplicity: the system was simple enough, the community case definition “any person who had unexplained illness with fever, diarrhoea, vomiting with or without bleeding which did not respond to antimalaria or who died after an unexplained severe illness with fever and bleeding” is also simple to understand. Few data were required to establish occurrence of EVD. The reporting form took less than 10 minutes to fill. However, simplicity was not 100% because the final confirmation required laboratory test.

**Stability:** the stability of the system was good. Notification forms were available at the health facilities. Staff and computers were available to run the system. System’s reliability (ability to collect, manage, and provide data without failure) and availability (ability to be operational when needed).

**Acceptability:** EVD was not part of the IDSR reporting system at the time of the evaluation. The level of acceptability was good. Five (5/6) of the interviewed health care workers were able to report the correct case definition and four (4/6) were able to give the correct procedure for specimen collection. Health workers were quite knowledgeable about case definition and specimen collection. The community were also willing to report cases to the health facility.

**Flexibility:** community case definition was introduced not to miss cases as it was in time past. This was easily done. Information technology were updated frequently even though data were entered on paper initially. Every alternate day the data from far chiefdoms were entered in batch. The EVD surveillance flexibility was excellent. When case definition were changed it was easy for the system to adapt. The participants’ willingness to accept changes was also commendable.

**Representativeness:** the level of representativeness was good. The reports were generated by all the active health facilities and communities. Facilities and communities without cases continued zero reporting for such period. Data were gathered from both private and public facilities. The data collection represented the state of EVD in the district. The feedback mechanism especially to the community was almost not in existence as evidenced by some community members reporting that they were not told whether or not their relative taken away by the ambulance survived or not.

**Sensitivity:** this refer to the proportion of cases of EVD detected by the surveillance system. Out of the 233 that had EVD status confirmatory test only 52 (21.1%) were positive. Table 2 was used to calculate the sensitivity and positive predictive value of the system. Sensitivity = 88.5% (46/52). The proportion of cases of EVD detected by the surveillance system is 88.5%.

**Table 2:** Symptoms using community definition and Laboratory diagnosis of EVD, (2 by 2 table) EVD Surveillance data Tonkolili District, Sierra Leone, December, 2014

Symptomatic Using Community definition	Laboratory confirmation of EVD		
	Yes	No	Total
Yes	46	132	178
No	6	49	55
**Total**	52	181	233

**Predictive value positive:** proportion of reported cases that have EVD. Out of all the reported cases by the system 25.8% (46/178) had EVD.

**Figure 3:**
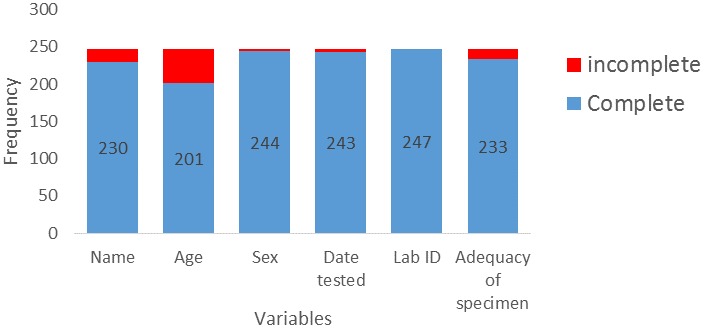
Completeness of EVD laboratory data, Tonkolili District Sierra Leone

**Data quality:** this refers to completeness and validity of the data recorded in the system. Data completeness was about 91%. Consistency exist in the data collected. In all December 2014 surveillance data was available for 247 individual. Figure 3 shows that 230 (93.1%) have their names in the records, age was documented for only 201(81.7%), sex 244(90.7%). Date tested was recorded for 243(98.4%). Specimen collected from the laboratory and field was adequate for testing in 233(94.3%). However, all the specimen were assigned laboratory identification number.

**Timeliness:** reflects the speed between steps of the system. Table 3 showed the day specimen got to the laboratory and day specimen was analysed. Only 31 (15.1%) of the samples got to the laboratory 2 or more days after collection. Delay in sending samples to the laboratory occurred at the community level. The target was to get the samples at most a day after collection. In all, 119 (50.6%) of the specimen were analysed the same day received. Specimen received late were analysed the following day. Speed was maintained in the system. The delay seen was from the collection of samples from the cases in the community.

**Table 3:** Day specimen got to the laboratory and day specimen was analysed, EVD Surveillance data Tonkolili District, Sierra Leone, December, 2014

Variables	Frequency	Percent
**Day specimen got to the Laboratory**		
Same day collected	106	51.7
Next day after collection	68	33.2
2 or more days	31	15.1
**Day specimen was analysed**		
Same day received	119	50.6
Next day	116	49.4

## Discussion

This study was conducted to evaluate the EVD surveillance system in Tonkolili District, Sierra Leone to determine the systems attributes and gaps requiring strengthening. The District Medical Officer of Health expressed that a major gap in the entire outbreak response was a dearth of personnel needed to support the epidemiology and surveillance team. Only few epidemiologist were in the district. The epidemiologist also added that more hands were needed for case investigation. The feedback to the community was also poor. Supportive supervision of contact tracers were required to facilitate effective surveillance system. Re-training of contact tracers was identified as a way to improve their activities, similar to a previous finding [13]. A community leader confirmed that more social mobilizers were needed in the communities. More community support was also encouraged. The Surveillance system reporting process was clear and easy as a result of frequent public awareness. Prior research has shown that for an effective and result-oriented surveillance system, it is pertinent to have a public awareness campaign to educate the affected community [14].

However, social mobilisation and community sensitization should be utilized as a key component because all stakeholders should be involved to enable them to pool resources and optimise the management of EVD cases and contacts [15]. The role community based intervention plays cannot be overemphasised [16]. The stability of the system was good. However, the stability was maintained through different international organisation and partners. Collaboration that would involve the local community more is needed. The health sector needs initiative towards strong workable partnerships with other sectors locally and internationally. This would not only produce the possible benefits of intersectoral synergy and efficiency but could also enhance the health status of the people [17]. Concerning acceptability the stakeholders were willing to participate in the system. Flexibility reflected the ability to adapt to changing information needs or technological operating conditions with little additional time, personnel and funds. Flexibility was demonstrated in the surveillance system.

Representativeness is the ability to accurately describe the occurrence of a health related event over time and its distribution in the population by place and person. The surveillance system was sensitive as reflected by the high proportion of cases detected. Out of all the reported cases by the system 25.8% had EVD. The PPV was low. A simplified but broad case definition was used at community level to alert HCWs or the appropriate level of the health system of a potential outbreak in the community. “any person who has unexplained illness with fever, diarrhoea, vomiting with or without bleeding which does not respond to antimalaria or who died after an unexplained severe illness with fever and bleeding”. Having a broad definition is good for the system. This also pointed to occurrence of other disease condition during the EVD outbreak [18]. Data completeness was more than 90%. Data quality was good while incomplete data and missing data was less than 10%. The good proportion of cases of EVD that was detected by the surveillance system was 88.5%.

However, it should be improved upon since the occurrence of a single case of EVD is an outbreak. The stability of the surveillance system was maintained through different international organisation and partners. The DHMT need to take ownership of the surveillance system for sustainability after the partners must have left. Diagnosis of other disease were not optimal because the EVD surveillance was not within the IDSR. Patients who could have had other diseases were tagged from the community as having EVD.

## Conclusion

EVD surveillance system was useful in providing data on trends and assessing progress of interventions. It provided information on the magnitude of morbidity and mortality due to EVD in the district. Data was therefore utilized for controlling the outbreak. **Limitation:** though the findings of this evaluation was used to improve the surveillance system the cost of running the system could not be ascertained due to the many stakeholders involved. **Recommendation:** restructuring of the data flow system to avoid delays and enhance reporting was recommended. More endorsement of the surveillance activities by chiefdom leadership was ensured. Communities were encouraged to be vigilant and immediately notify any clusters of an unusual illnesses or deaths occurring in their communities either through the Ebola Help line, or directly to the DHMT. **Public health actions:** the findings of the evaluation was discussed with District Medical Officer and other stakeholders. Presentation was done at the epidemiology and surveillance pillar meeting. Training of contact tracers was done to close all identified gaps. More Sierra Leone indigenes were added to the programme. Follow up actions were planned by re-evaluation of available surveillance data for the subsequent month and check differences in the attributes.

### What is known about this topic

- Surveillance activities are important to bringing an end to infectious disease outbreak;
- Considering the importance of Public health surveillance systems, evaluation should be conducted periodically;
- The purpose of evaluating public health surveillance systems is to ensure that problems of public health importance are being monitored efficiently and effectively.

### What this study adds

- EVD was not part of the routine Integrated Disease Surveillance and Response (IDSR) in Sierra Leone prior to the 2013 outbreak;
- Findings helped to restructure the data flow system to avoid delays and enhance reporting;
- Training was conducted for contact tracers.

## Competing interests

The authors declare no competing interests.
